# Diffusion-Weighted MR Imaging of the Thymus in Children with Non-Thymic Neoplasms

**DOI:** 10.3390/diagnostics13243654

**Published:** 2023-12-13

**Authors:** Sook Min Hwang, Ji Eun Park, So-Young Yoo, Ji Hye Kim, Sun-Young Baek, Sung-Hoon Moon, Tae Yeon Jeon

**Affiliations:** 1 Department of Radiology, Hallym University Kangnam Sacred Heart Hospital, Seoul 07441, Republic of Korea; 2 Department of Radiology and Center for Imaging Science, Samsung Medical Center, Sungkyunkwan University School of Medicine, Seoul 06531, Republic of Korea; je2102.park@samsung.com (J.E.P.);; 3 Biomedical Statistics Center and Research Institute for Future Medicine, Samsung Medical Center, Sungkyunkwan University School of Medicine, Seoul 06351, Republic of Korea; 4 Department of Internal Medicine, Hallym University Sacred Heart Hospital, Anyang 14068, Republic of Korea

**Keywords:** thymus, diffusion-weighted imaging, apparent diffusion coefficient, pediatrics

## Abstract

**Purpose:** To investigate the diffusion-weighted imaging (DWI) findings and apparent diffusion coefficient (ADC) value of the thymus in children under the age of 13 who have non-thymic neoplasms. **Materials and Methods**: From January 2021 to April 2023, a single-center retrospective study analyzed consecutive 191 thoracic MRI scans with DWI from 67 children (<13 years) with non-thymic neoplasms. The scans were categorized based on the presence of restricted diffusion on DWI. We analyzed the demographics, laboratory data, and MR findings of the thymus. Statistical differences were assessed using generalized estimating equations. **Results**: Forty-five percent (86/191) of the scans exhibited restricted diffusion in the thymus: multifocal (*n* = 65; 76%), focal (*n* = 19; 22%), and diffuse (*n* = 2; 2%) patterns. The restricted diffusion group demonstrated higher absolute lymphocyte counts, more prominent thymus sizes, and higher frequency of off-treatment status compared to the unrestricted diffusion group (*p*>0.05). The mean ADC value across all 191 scans was 1.80 × 10^−3^ mm^2^/s. No significant difference was observed in the ADC value related to restricted diffusion patterns, the MRI vendors, or the age at the time of the scan. **Conclusions**: Restricted diffusion was observed in 45% of the thymus in children with non-thymic neoplasms, with a mean ADC value of 1.80 × 10^−3^ mm^2^/s. Recognizing the DWI findings can prevent unnecessary invasive procedures, alleviating concerns for patients and families.

## 1. Introduction

Diffusion-weighted imaging (DWI) offers both functional and structural insights into biological tissues without the need for ionizing radiation or intravenous contrast. It measures the mobility of water molecules, reflecting the cellular density of tissues [1,2]. DWI can differentiate between malignant and benign conditions through assessing the apparent diffusion coefficient (ADC) values both qualitatively and quantitatively. Malignancies typically display high-signal intensities on DWIs and are accompanied by low-signal intensities on ADC maps [1,2].

The thymus is a crucial lymphatic organ, essential for the development of the adaptive immune system. It is most active during the neonatal and pre-adolescent periods [3,4,5]. Starting in early adolescence, the size and activity of the thymus diminish, with the thymic tissue progressively being replaced with adipose tissue [4,6,7,8].

In adults, the normal thymus, characterized by low cellularity and high fat content, can be differentiated from thymic tumors by showing no restricted diffusion on DWIs and signal suppression on opposed-phase images [9,10,11]. Conversely, the pediatric thymus, which has more cells and less fat, yields varied MR imaging findings, potentially leading to diagnostic confusion [4,6,7,8]. While chemical-shift MR can detect as low as 10% lipid content, the normal pediatric thymus often does not exhibit signal suppression on opposed-phase images [9,12]. It is important to note that the pediatric thymus may exhibit restricted diffusion on DWIs [13,14]. Hence careful attention is required to avoid mistaking a normal thymus showing restricted diffusion for thymic tumors in children who are undergoing follow-ups for thoracic non-thymic neoplasms that can display restricted diffusion. Comprehensive DWI findings specific to the normal thymus in children are still limited.

The purpose of this study was to investigate the DWI findings and ADC value of the thymus in children under the age of 13 who have non-thymic neoplasms, following a minimum of 6 months of clinical and imaging follow-up.

## 2. Materials and Methods

### 2.1. Study Population

This retrospective study was approved by the institutional review board of Samsung Medical Center (2023-10-119). The requirement for informed consent was waived, and all patient data were anonymized and de-identified before analysis.

We conducted a retrospective search of medical records from January 2021 to April 2023 using our electronic database, identifying 230 thoracic MRI scans with DWIs for 88 children. All patients underwent these MRIs due to their underlying thoracic non-thymic neoplasms. Subjects under 13 years of age were selected due to age-related thymic involution. From this cohort, 39 thoracic MRI scans were excluded for the following reasons: (a) presence of neoplastic conditions affecting the thymus (*n* = 1); (b) thymus was not visible on thoracic MRI (*n* = 10); (c) difficulty in evaluating the thymus due to artifacts (*n* = 9); and (d) absence of a DWI (*n* = 19). Consequently, our study comprised 191 thoracic MRI scans from 67 children. To verify that these cases of restricted diffusion were not linked to pathological conditions, our study employed a clinical and imaging follow-up period of six months or longer.

The medical records of the eligible patients were reviewed to collect demographic and laboratory data within two weeks of their thoracic MRI scans. The demographics included the age at the time of the MRI examination, sex, the underlying disease prompting the MRI, and treatment status during the MRI (on-treatment vs. off-treatment). On-treatment status was defined as cases where the MRI was conducted within one week following chemotherapy, radiation therapy, or surgery. Conversely, off-treatment status referred to cases over one week post-treatment (chemotherapy, radiation therapy, or surgery), before treatment initiation, or under observation without treatment during the MRI.

Laboratory data included WBC counts, absolute neutrophil counts, absolute lymphocyte counts, and platelet counts.

### 2.2. Image Analysis

Two radiologists (T.Y.J. and S.M.H., with 14 and 9 years of pediatric thoracic imaging interpretation experience, respectively), who were blinded to the patient’s clinical information, reviewed all of the MR images by consensus.

The MRI features of the thymus were assessed based on the following: (a) thymus signal intensity compared to muscle on T2- and T1-weighted images; (b) thymus size on T2-weighted image (diminished vs. prominent); (c) the presence or absence of the restricted diffusion; and (d) the pattern of the restricted diffusion (multifocal, focal, and diffuse).

A diminished thymus was defined by a thickness of the thymus less than 2 cm on an axial scan [15]. The presence of restricted diffusion in the thymus was identified through signal intensity suppression at high b-value (b = 800) DWI and corresponding low ADC values on ADC maps. The ADC value of the thymus was quantitatively measured using the largest possible circular or oval region-of-interest (ROI; range: 9.2–221.9 mm^2^). Three ROIs were manually drawn on consecutive ADC images, and the average of these three ROI measurements was used for the statistical analysis to minimize errors. The ROIs were defined by selecting regions with hyperintensity on high b-value (b = 800 mm^2^/s) DWI and relatively low ADC values. Care was taken to avoid adjacent lung tissue, major blood vessels, the heart, and any artifacts. The illustration of ROI placement for the ADC measurement of the thymus is shown in the Supplementary Figure S1.

In patients undergoing follow-up MRI examinations, changes in restricted diffusion status and its pattern in the thymus were evaluated. Additionally, we investigated whether changes in restricted diffusion status or restricted diffusion pattern were associated with changes in MRI vendor, thymus size, or treatment status.

### 2.3. Imaging Techniques

MRI studies were conducted using 3.0 T units from three vendors: Philips (Achieva, Philips Healthcare, Best, the Netherlands; *n* = 84, 44%), Siemens A (MAGNETOM Skyra, Siemens Healthineers, Erlangen, Germany; *n* = 60, 31%), and Siemens B (MAGNETOM Vida, Siemens Healthineers, Erlangen, Germany; *n* = 47, 25%). The MRI protocol consisted of axial and coronal T2-weighted images, axial T1-weighted images, and DWIs, all acquired using 2-dimensional single-slice techniques. The MR imaging parameters are described in Table 1. We regularly employed electrocardiography gating and, when required, applied respiratory compensation to achieve superior and reliable image quality. All ADC values were computed using a mono-exponential function with b-values of 0 and 800 mm^2^/s.

Before the examination, patients were required to fast for a minimum of 3 h. Where feasible, MR examinations were conducted during natural sleep or while awake. If necessary, sedatives were provided by a skilled pediatric sedation team upon parental consent. Out of 191 MRI examinations, sedatives were used for 126 examinations (mean age at the time of MRI was 3.5 years, ranging from 0.1 to 8 years).

### 2.4. Statistical Analysis

All statistical analyses were performed using SAS statistical software (version 9.4; SAS Institute Inc., Cary, NC, USA). Statistical significance was set at *p* < 0.05. Generalized estimating equations were applied to differentiate between variables for the restricted diffusion and unrestricted diffusion groups. Additionally, correlations between the ADC value and three different restricted diffusion patterns, between the ADC value and three different MRI vendors, and between the ADC value and the age at the MRI examination were explored. Generalized estimating equations were employed to address the concern of repeated measurement variables.

## 3. Results

The baseline characteristics of the study population are detailed in Table 2. The most common underlying disease was mediastinal neuroblastoma, which constituted 49% (33/67) of all cases.

**Figure 1:**
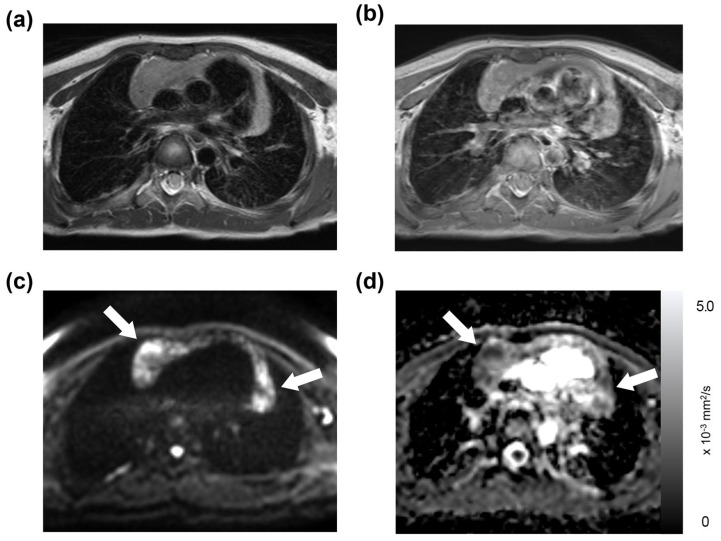
Multifocal restricted diffusion in the thymus: a 6-year-old boy with a history of mediastinal ganglioneuroblastoma. This patient underwent an MRI on a Philips scanner, with their laboratory data showing a WBC count of 5.08 × 10^3^/μL, an absolute neutrophil count of 1.97 × 10^3^/μL, an absolute lymphocyte count of 2.42 × 10^3^/μL, and a platelet count of 288 × 10^3^/μL. The thymus was found to be slightly hyperintense on both the T2-weighted (**a**) and T1-weighted images (**b**). Multifocal restricted diffusion was present on the DWI (**c**, arrows), which corresponds to low-signal intensity in the ADC map with a mean ADC value of 1.17 × 10^−3^ mm^2^/s (**d**, arrows).

**Figure 2:**
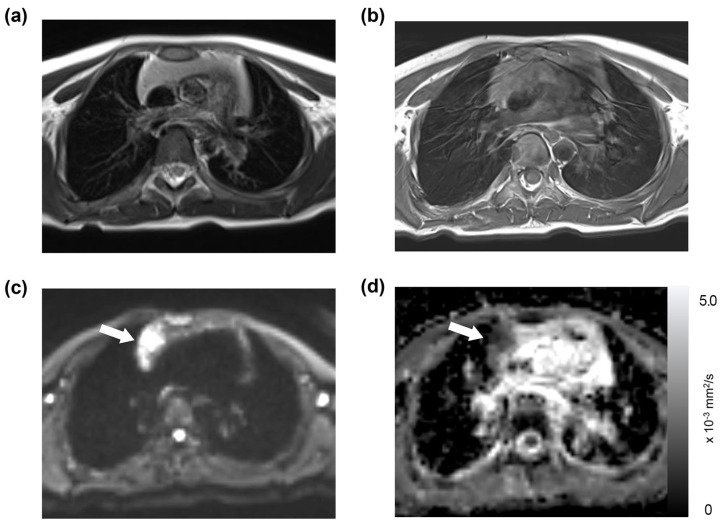
Focal restricted diffusion in the thymus: a 5-year-old girl with a history of mediastinal ganglioneuroblastoma. This patient underwent an MRI on a Siemens B scanner, with their laboratory data showing a WBC count of 8.13 × 10^3^/μL, an absolute neutrophil count of 2.89 × 10^3^/μL, an absolute lymphocyte count of 3.94 × 10^3^/μL, and a platelet count of 216 × 10^3^/μL. The thymus was found to be slightly hyperintense on the T2-weighted image (**a**) and isointense on the T1-weighted image (**b**). Focal restricted diffusion was present on the DWI (**c**, arrow), correlating with low-signal intensity in the ADC map with a mean ADC value of 1.04 × 10^−3^ mm^2^/s (**d**, arrow).

**Figure 3:**
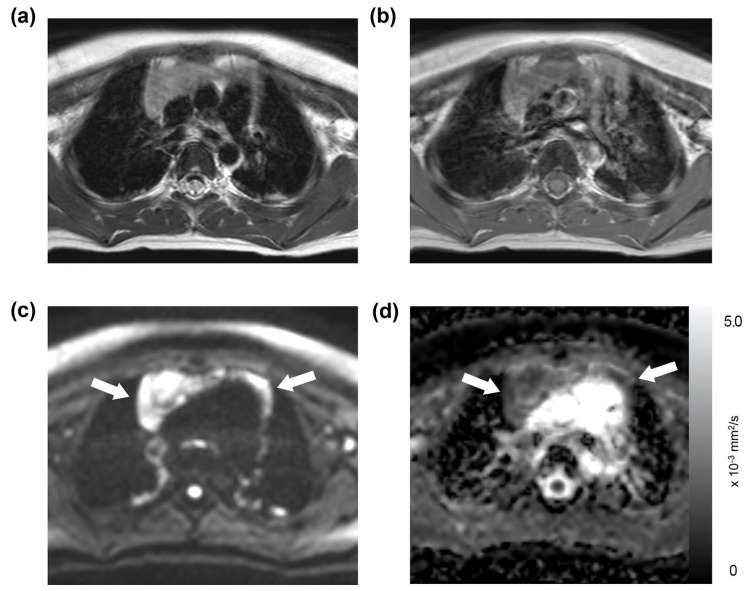
Diffuse restricted diffusion in the thymus: a 4-year-old boy with a history of mediastinal neuroblastoma. This patient underwent an MRI on a Siemens A scanner, with their laboratory data showing a WBC count of 8.43 × 10^3^/μL, an absolute neutrophil count of 4.37 × 10^3^/μL, an absolute lymphocyte count of 3.15 × 10^3^/μL, and a platelet count of 215 × 10^3^/μL. The thymus was found to be slightly hyperintense on the T2-weighted image (**a**) and isointense on the T1-weighted image (**b**). Diffuse restricted diffusion was present on the DWI (**c**, arrows), which corresponds to low-signal intensity in the ADC map with a mean ADC value of 1.23 × 10^−3^ mm^2^/s (**d**, arrows).

**Figure 4:**
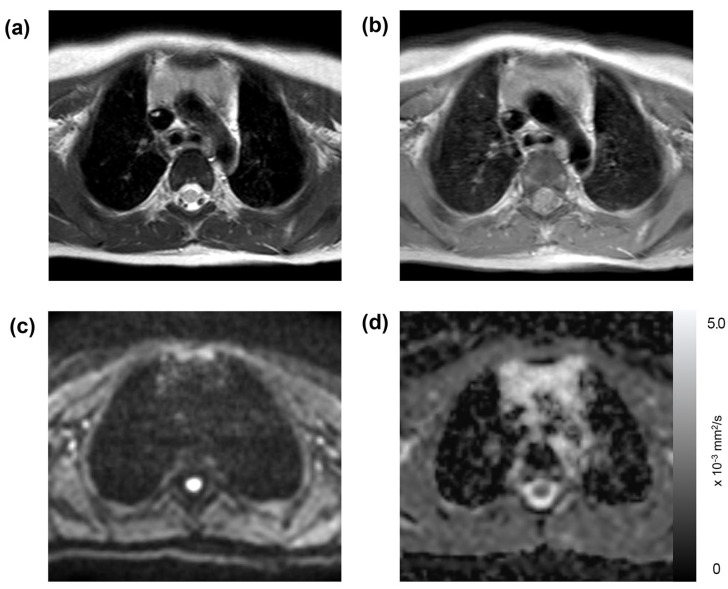
Unrestricted diffusion in the thymus: a 1-year-old boy with a history of mediastinal neuroblastoma. This patient underwent an MRI on a Philips scanner, with their laboratory data showing a WBC count of 1.22 × 10^3^/μL, an absolute neutrophil count of 0.67 × 10^3^/μL, an absolute lymphocyte count of 0.23 × 10^3^/μL, and a platelet count of 169 × 10^3^/μL. The thymus was found to be slightly hyperintense on both the T2-weighted (**a**) and T1-weighted images (**b**). A DWI with a b-value of 800 s/mm^2^ (**c**, “White dot” refers to a normal structure (normal spinal cord) that is unrelated to the lesion) exhibits hypointensity and no reduction in the ADC map with a mean ADC value of 1.96 × 10^−3^ mm^2^/s (**d**).

Of the 191 thoracic MRI scans, restricted diffusion in the thymus was identified in 86 studies (45%), affecting 40 patients; this group was referred to as the “restricted diffusion group” (Figure 1, Figure 2 and Figure 3). The remaining 105 studies (55%), encompassing 50 patients, exhibited no restricted diffusion in the thymus and were designated the “unrestricted diffusion group” (Figure 4). The restricted diffusion group exhibited three different restricted diffusion patterns: multifocal (*n* = 65; 76%) (Figure 1), focal (*n* = 19; 22%) (Figure 2), and diffuse (*n* = 2; 2%) (Figure 3).

Comparisons between the restricted diffusion and unrestricted diffusion groups are presented in Table 3. Variables like age, sex, underlying disease, and MRI vendor did not significantly differ between the groups. Laboratory data were obtained within two weeks of 187 MRI scans for 63 patients. The absolute lymphocyte count was found to be significantly higher in the restricted diffusion group (median: 2.74; interquartile range: 2.14–3.67) than in the unrestricted diffusion group (median: 2.01; interquartile range: 0.60–2.63) (*p* < 0.001). Regarding the T2-weighted images, the thymus consistently appeared with a homogeneous appearance and was slightly hyperintense relative to the muscle in all 191 MRI scans. In terms of the T1-weighted images, the thymus was either slightly hyperintense (*n* = 58; 30%) or isointense (*n* = 131; 70%) relative to the muscle, with no significant differences between the restricted diffusion and unrestricted diffusion groups (*p* = 0.171). The diminished thymus was more common in the unrestricted diffusion group (7% (6/86) vs. 52% (55/105)), while prominent thymuses were frequently identified in the restricted diffusion group (93% (80/86) vs. 48% (50/105)) (*p* < 0.001). The restricted diffusion group was predominantly characterized by off-treatment status (94% (81/86) vs. 60 (63/105)), whereas the on-treatment status was more common in the unrestricted diffusion group (6% (5/86) vs. 40% (42/105)) (*p* < 0.001). Treatment status subtypes were as follows: chemotherapy (*n* = 41; 21%), radiation therapy (*n* = 3; 2%), surgery (*n* = 3; 2%), post-treatment (*n* = 110; 58%), before treatment initiation (*n* = 19; 10%), and under observation without treatment (*n* = 15; 8%).

**Figure 5:**
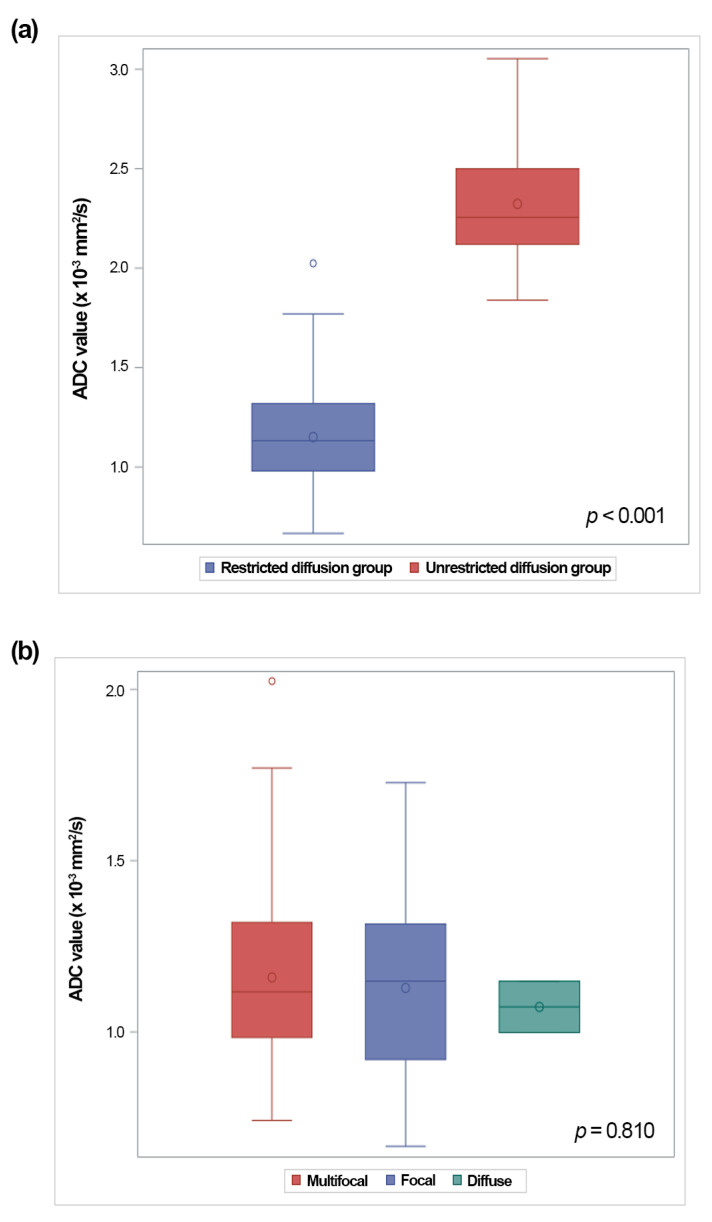
Graphs showing the ADC values of the thymus for the restricted diffusion group versus the unrestricted diffusion group (**a**) and for the three patterns of restricted diffusion (**b**). The boxes represent the interquartile range (25th to 75th percentile), with the median marked by the horizontal line inside the boxes. The whiskers extend to the minimum and maximum values within 1.5 times the interquartile range of the lower and upper quartiles, respectively. The small circle inside the boxes represents the average value. The value outside this range is displayed as an individual point. (**a**) There is a significant difference in the ADC values between the two groups (*p* < 0.001). (**b**) There is no significant difference in the ADC values among the three patterns of restricted diffusion (*p* = 0.810).

**Figure 6:**
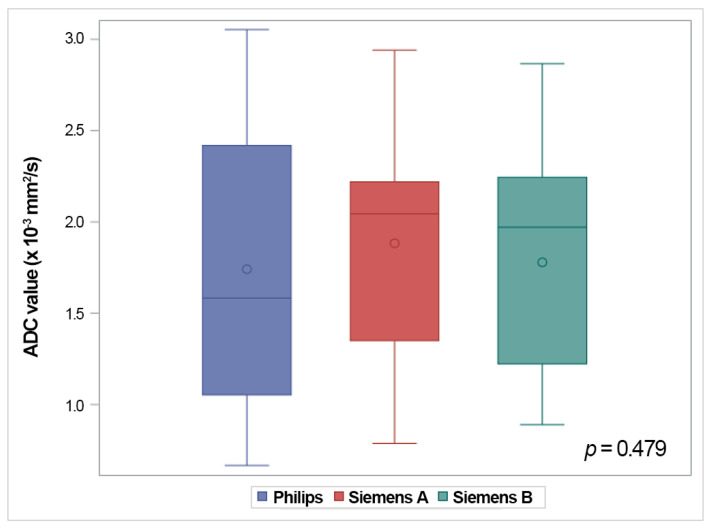
Graphs showing the ADC values of the thymus for the three different MRI vendors. The boxes represent the interquartile range (25th to 75th percentile), with the median marked by the horizontal line inside the boxes. The whiskers extend to the minimum and maximum values within 1.5 times the interquartile range of the lower and upper quartiles, respectively. The small circles inside the boxes represent the average value. The ADC values do not differ significantly between the MRI vendors (*p* = 0.479).

The mean ADC value of the thymus in all 191 studies was 1.80 × 10^−3^ mm^2^/s (range: 0.67–3.05 × 10^−3^ mm^2^/s). The ADC values for both groups, for the three patterns of restricted diffusion, and for the three different MRI vendors are represented with box-and-whisker plots in Figure 5 and Figure 6. The restricted diffusion group exhibited significantly lower ADC values (median: 1.13 × 10^−3^ mm^2^/s; interquartile range: 0.98–1.32 × 10^−3^ mm^2^/s) compared to the unrestricted diffusion group (median: 2.26 × 10^−3^ mm^2^/s; interquartile range: 2.12–2.50 × 10^−3^ mm^2^/s) (*p* < 0.001) (Figure 5a). The median ADC value was 1.12 × 10^−3^ mm^2^/s (interquartile range: 0.98–1.32 × 10^−3^ mm^2^/s) for the multifocal pattern, 1.15 × 10^−3^ mm^2^/s (interquartile range: 0.92–1.31 × 10^−3^ mm^2^/s) for the focal pattern, and 1.07 × 10^−3^ mm^2^/s (interquartile range: 1–1.15 × 10^−3^ mm^2^/s) for the diffuse pattern (Figure 5b). No significant differences were observed between the ADC values for the thymus and the three patterns of restricted diffusion (*p* = 0.810). The median ADC value was 1.58 × 10^−3^ mm^2^/s (interquartile range: 1.05–2.42 × 10^−3^ mm^2^/s) for the Philips, 2.04 × 10^−3^ mm^2^/s (interquartile range: 1.35–2.22 × 10^−3^ mm^2^/s) for the Siemens A, and 1.97 × 10^−3^ mm^2^/s (interquartile range: 1.22–2.24 × 10^−3^ mm^2^/s) for the Siemens B. No significant differences in the ADC values were found among the three different MRI vendors (*p* = 0.479) (Figure 6). No correlations were detected between the ADC values and the age at the MRI examination (*p* = 0.798).

**Figure 7:**
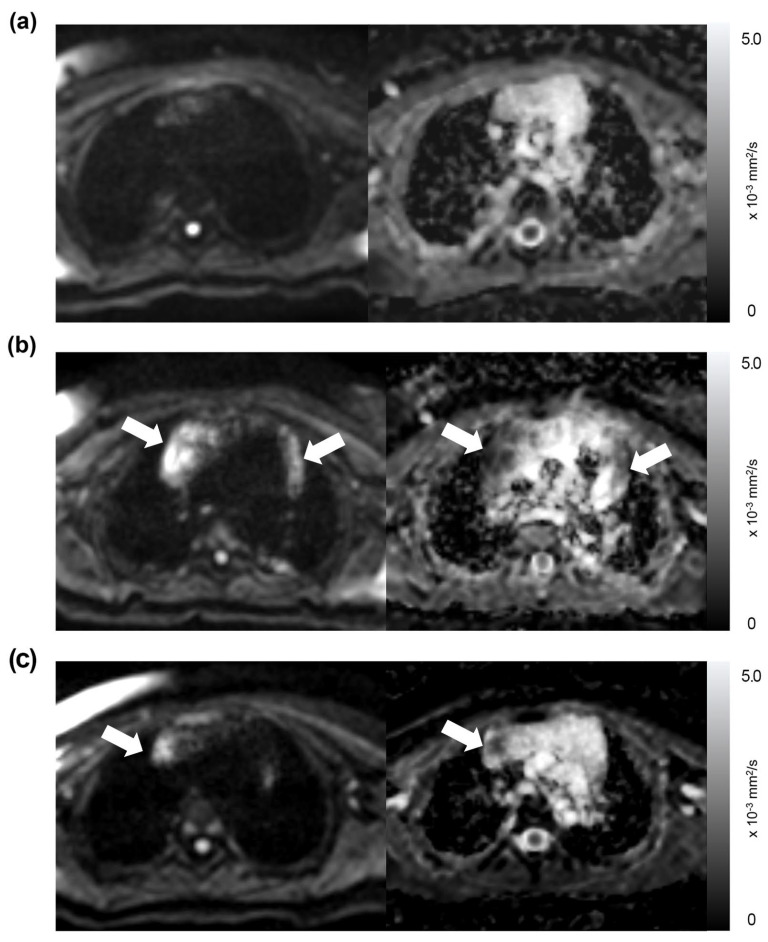
Changes in the presence and pattern of restricted diffusion observed in follow-up MRIs of a girl with a history of mediastinal neuroblastoma. (**a**) The MRI performed when the girl was 17 months old showed the thymus exhibiting unrestricted diffusion (Philips MRI, treatment status: on-treatment (chemotherapy), and thymus size: diminished) (**b**) The next MRI, performed 4 months later, revealed a multifocal pattern (arrows) of restricted diffusion (Siemens A MRI, treatment status: off-treatment (post-treatment), and thymus size: prominent). (**c**) Another follow-up MRI, performed 8 months after the second one, showed a focal pattern (arrow) of restricted diffusion (Philips MRI, treatment status: off-treatment (post-treatment), and thymus size: prominent).

**Figure 8:**
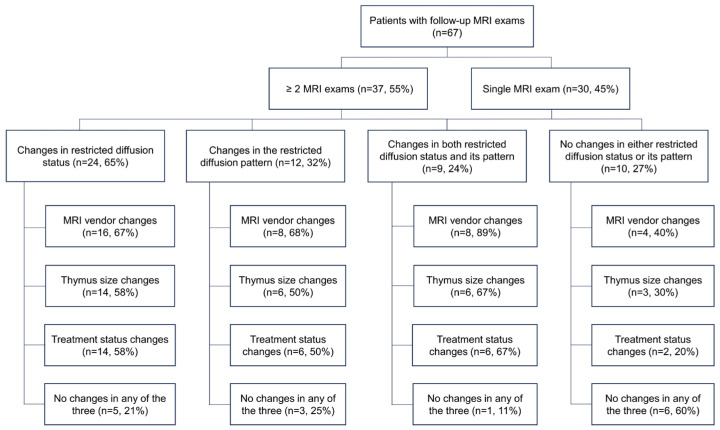
Changes in the restricted diffusion status and its pattern in the thymus during follow-up MRI examinations. The term ‘no changes in any of the three’ means that there were no alterations in the MRI vendor, thymus size, and treatment status.

Of the 67 patients, 37 underwent more than two MRI examinations, with an average of 4.4 scans (range: 2–10 times). Among these patients, 24 (65%) exhibited changes in restricted diffusion status, and 12 (32%) in the diffusion restriction pattern during follow-up MRIs. Nine patients (24%) displayed changes in both, as illustrated in Figure 7, while 10 (27%) showed no changes in either. Changes in the restricted diffusion status and its pattern in the thymus observed during follow-up MRI examinations are summarized in Figure 8.

## 4. Discussion

The present study revealed that 45% of the pediatric thymus with non-thymic neoplasm exhibits restricted diffusion on DWIs, manifesting in one of three patterns: multifocal (76%), focal (22%), and diffuse (2%). Our study is the first, to our knowledge, to evaluate the incidence and pattern of restricted diffusion of the pediatric thymus with non-thymic neoplasm on DWIs. The mean ADC value in the pediatric thymus aged below 13 years old was 1.80 × 10^−3^ mm^2^/s, with the restricted diffusion group showing 1.15 × 10^−3^ mm^2^/s and the unrestricted diffusion group’s being 2.32 × 10^−3^ mm^2^/s. Our study also demonstrated the conditions associated with a restricted diffusion of the thymus. Elevated absolute lymphocyte count, increased thymus size, and off-treatment status commonly accompanied restricted diffusion of the thymus in children.

From the time of birth, the thymus undergoes a gradual growth process, reaching its peak weight during the neonatal and pre-adolescent stages [3,4,5]. Starting from the early teens, the thymus begins to decrease in size. This reduction is marked by a gradual replacement of glandular tissue with fatty tissue, alongside the disruption of epithelial tissue and an arrest in thymocyte development [4,6,7,8,16]. By around the age of 40 years, the thymus is typically composed of fatty tissue almost entirely, leaving only about 5% of the original thymic tissue intact [17]. In infants, the thymus has a quadrilateral shape, which later transforms into a triangular or arrowhead-like shape [8,18,19].

In young individuals, an enlarged thymus can be misinterpreted as mediastinal tumors in childhood [20,21]. Differentiating a normal thymus from mediastinal tumors solely based on morphologic assessment can be challenging, given the wide variability in the size and shape of the thymus [9,22,23]. In adolescents and young adults, the normal thymus can show attenuation values on CT scans and signal intensities on MRI scans that resemble those of mediastinal tumors [8,18,19,24,25]. This is due to the fact that the thymus has not yet been fully replaced by fatty tissue. Consequently, chemical-shift MRI is less effective in children, as their thymuses contain minimally intercalated fat [9,12]. A study of 95 healthy subjects showed that chemical shift imaging can detect fatty infiltration of the thymus for all patients over 15 years, nearly half of patients aged 11–15 years, but no patients aged 10 years or younger, although microscopic amounts of fats are present from birth [12]. Hence, DWI may be useful for characterizing a normal thymus in those under 15 years of age, as it displays unrestricted diffusion on DWIs with high ADC values when chemical shift imaging shows no signal loss [9].

No study has yet fully assessed the ability of DWI to characterize the normal pediatric thymus in healthy subjects, although there are some limited data available. Only two cases have demonstrated restricted diffusion in the ectopic cervical thymus of boys aged 1 and 2 months, without measuring the ADC value of the thymus [13,14]. In a study of 87 myasthenia gravis cases aged 15–71 years, the mean ADC value for the normal thymus was 2.1 × 10^−3^ mm^2^/s. This was significantly different from lymphoid thymic hyperplasia (with a mean ADC of 1.86 × 10^−3^ mm^2^/s) and thymoma (with a mean ADC of 1.36 × 10^−3^ mm^2^/s) [26]. In another study involving subjects aged 9–23 years, the lipid-poor thymus displayed unrestricted diffusion, with a mean ADC value of 2.48 × 10^−3^ mm^2^/s, and it also revealed a positive correlation between the patient’s age and the ADC, with the youngest subjects having the lowest ADC [9]. This indicates a decrease in thymus cellular density with age, due to the progressive replacement with fats. In our study, which focused on patients under 13 years of age, the mean ADC was 1.8 × 10^−3^ mm^2^/s, lower than reported in previous studies, with 45% of the subjects commonly exhibiting restricted diffusion of the thymus, possibly due to higher cellularity at a younger age [4,6,7,8].

The thymus varies widely in appearance during childhood, even within the same individual. Apart from the age-related thymic involution, thymus size may also reduce in about 90% of cases during treatments, such as chemotherapy and radiation therapy [27]. However, during the recovery phase after these treatments for neoplasms, the thymus often regrows. Additionally, thymic hyperplasia, which is an enlargement of the thymus, can occur as a rebound phenomenon. This is relatively common, occurring in approximately 25% of cases [27,28]. In our study, we found that a prominent thymus size and off-treatment status were often linked to restricted diffusion of the thymus, possibly due to rebound thymic hyperplasia. The restricted diffusion of the rebound thymic hyperplasia may be due to increased cellular density from an increased number of thymic epithelial cells and lymphoid germinal centers [29]. Furthermore, our study showed a correlation between a higher absolute lymphocyte count and restricted diffusion of the thymus, suggesting that thymic hyperplasia might manifest as an increase in peripheral blood lymphocytes [30]. The thymus plays an essential role in the maturation of T-lymphocytes. However, the implications of its size or changes in its size on the evolving immune system have yet to be fully understood. Several studies have investigated the association between peripheral blood lymphocyte counts and thymus size during infancy. One study on 31 HIV-infected children identified a positive correlation between the thymus size and the number of T-lymphocyte subsets, especially CD4+ cells [31]. Furthermore, a sonographic study by Jeppesen et al. [32] on breastfed infants indicated a correlation between the thymus size and the count of certain T-lymphocyte subsets (CD4+ and CD8+ cells) in the peripheral blood. These observations imply that an increase in thymus size, as seen in rebound thymic hyperplasia, could potentially lead to lymphocytosis.

Our study documented alterations in the restricted diffusion status and its pattern within the thymus among patients undergoing follow-up MRI exams (as shown in Figure 8). Changes in the diffusion status or pattern may be linked to changes in the MRI vendor, alterations in the thymus size, and shifts in the treatment status. We have presented these findings in a descriptive manner, as no suitable statistical methodology was available for our analyses.

Our results may be analogous to those observed with PET-CT. According to Jerushalmi et al. [33], physiologic thymic uptake of ^18^F-fluorodeoxyglucose was observed in 28% of the study population comprising children and young adults. Notably, thymic uptake was found in 73% of untreated children aged 13 years or younger. Physiologic thymic activity should be taken into considerations when evaluating functional imaging modalities, such as PET-CT and diffusion-weighted MR imaging.

This study has several limitations. It was based on a retrospective analysis at a single institution. While we lacked histopathological confirmation for diagnosing a normal thymus, we validated the thymus without non-thymic neoplasm based on clinical and imaging follow-ups. It is important to underscore that our study population consisted of individuals with non-thymic neoplasms, rather than healthy volunteers. Consequently, assessing the normal thymus in healthy individuals may not yield results congruent with our data. Follow-up studies may be necessary to assess restricted diffusion in the true normal thymuses of healthy controls. In our study, we assessed thymus ADC values using three different 3.0 T MRI systems. Significant inter-vendor variations in ADC values can be influenced by patient factors, hardware, sequence-related factors, such as different b-values or breathing protocols, and artifacts from susceptibility effects to eddy currents [34,35,36]. However, Donati et al.’s study [37], which focused on the upper abdominal organs, found their ADC values to be comparable across various MR systems and field strengths, even though it did not specifically examine the thymus. Finally, the ROI was determined by a single observer, and we did not evaluate inter-observer agreement. Nevertheless, in our study, the ROIs were consistently placed on the largest slice of the clearly delineated thymus, making the ROI analysis less subject to observer bias.

In conclusion, our study revealed that the normal pediatric thymus can exhibit restricted diffusion in three distinct patterns: multifocal, focal, or diffuse. This was particularly observed in cases with elevated absolute lymphocyte counts, prominent thymus sizes, and in patients with off-treatment status. Our results will likely help establish a reference for the ADC value in the thymuses of children with thoracic non-thymic neoplasms. Further research is needed to validate the significance of ADC values in distinguishing normal thymuses from thymic pathology in children. Being familiar with the characteristic DWI findings and mean ADC value for the pediatric thymus may potentially minimize invasive procedures and alleviate concerns of both patients and their families.

## Figures and Tables

**Table 1:** Thoracic MRI protocol.

	Philips				Siemens A				Siemens B			
	DWI	Axial T2-WI	Coronal T2-WI	Axial T1-WI	DWI	Axial T2-WI	Coronal T2-WI	Axial T1-WI	DWI	Axial T2-WI	Coronal T2-WI	Axial T1-WI
Imaging technique	EPI	TSE	TSE	TSE	EPI	BLADE	BLADE	TSE	EPI	BLADE	BLADE	TSE
B-values (mm^2^/s)	0 and 800				0 and 800				0 and 800			
TR/TE (ms)	1672/60	2118/65	2118/65	700/5	7500/56	3690/95	5160/95	100/8.4	7300/64	3710/91	2760/90	413/8.4
Flip angle (°)	90	90	90	90	90	106	102	120	90	113	113	120
NEX	2	2	2	1	2 and 8	1	1	1	2 and 4	1	1	1
Matrix size	192 × 192	512 × 512	512 × 512	512 × 512	260 × 260	380 × 380	380 × 380	380 × 380	360 × 360	360 × 360	400 × 400	360 × 360
FOV (mm)	260	250	250	250	340	320	320	320	360	360	400	360
Slice thickness (mm)	5	5	5	5	5	5	4	5	5	5	4	5
Acquisition time (min:s)	1:30	5:18	3:15	2:20	1:42	1:30	1:10	1:40	2:04	1:40	00:50	01:22

Abbreviations: DWI—diffusion-weighted imaging, T2-WI—T2-weighted imaging, T1-WI—T1-weighted imaging, EPI—echo-planar imaging, TSE—turbo spine echo, TR—repetition time, TE—echo time, NEX—number of excitations, and FOV—field of view.

**Table 2:** Baseline characteristics of 67 children who underwent 191 thoracic MRI scans.

Variable	Patients (*N* = 67)
Age (y) ^a,b^	5.5 ± 3.7
Male-to-female ratio	32:35
Underlying disease ^c^	
Neuroblastoma	33 (49%)
Ganglioneuroblastoma	18 (27%)
Congenital vascular malformation	9 (13%)
Ganglioneuroma	2 (3%)
Undifferentiated pleomorphic sarcoma	1 (1%)
Pulmonary sequestration	1 (1%)
Duplication cyst	1 (1%)
Ewing sarcoma	1 (1%)
Lipoblastoma	1 (1%)

^a^ Data are presented as the mean ± standard deviation. ^b^ The patient’s age was taken from the first MRI scan. ^c^ Data are presented as the numbers of patients, with percentages in parentheses.

**Table 3:** Comparisons between the restricted diffusion and unrestricted diffusion groups in 191 thoracic MRI scans.

Variable	Total	Restricted Diffusion (*n* = 86)	Unrestricted Diffusion (*n* = 105)	*p*-Value
Age (y) ^a^	4.8 (2.5, 8.4)	4.8 (2.6, 8.4)	4.8 (2.3, 8.4)	0.777
Sex, male	81 (42%)	37 (43%)	44 (42%)	0.897
Underlying disease				0.842
Benign	19 (10%)	9 (10%)	10 (10%)	
Malignant	172 (90%)	77 (90%)	95 (90%)	
MRI vendor				0.258
Philips	84 (44%)	43 (50%)	41 (39%)	
Siemens A	60 (31%)	21 (24%)	39 (37%)	
Siemens B	47 (25%)	22 (26%)	25 (24%)	
Laboratory data ^a,b^				
WBC count (×10^3^/μL)	5.41 (4.08, 6.67)	5.63 (4.56, 6.96)	5.21 (3.41, 6.38)	0.130
Absolute neutrophil count (×10^3^/μL)	2.2 (1.6, 3.1)	2.2 (1.65, 2.89)	2.2 (1.59, 3.4)	0.144
Absolute lymphocyte count (×10^3^/μL)	2.36 (0.91, 3.16)	2.74 (2.14, 3.67)	2.01 (0.60, 2.63)	<0.001
Platelet count (×10^3^/μL)	250 (198, 314)	251 (215, 304)	241 (164, 317)	0.530
Thymus size				<0.001
Diminished	61 (32%)	6 (7%)	55 (52%)	
Prominent	130 (68%)	80 (93%)	50 (48%)	
Thymus signal intensity				
Slightly hyperintense on T2-WI	191 (100%)	86 (100%)	105 (100%)	1
Slightly hyperintense on T1-WI	58 (30%)	31 (36%)	27 (26%)	0.171
Isointense on T1-WI	133 (70%)	55 (64%)	78 (74%)	
Treatment status ^c^				<0.001
On-treatment	47 (25%)	5 (6%)	42 (40%)	
Non-treatment	144 (75%)	81 (94%)	63 (60%)	
Treatment status subtype ^c^				
Chemotherapy	41 (21%)	3 (3%)	38 (36%)	<0.001
Radiation therapy	3 (2%)	1 (1%)	2 (2%)	0.571
Surgery	3 (2%)	1 (1%)	2 (2%)	0.571
Post-treatment	110 (58%)	65 (76%)	45 (43%)	0.493
Before treatment initiation	19 (10%)	8 (9%)	11 (10%)	0.808
Under observation without treatment	15 (8%)	8 (9%)	7 (7%)	0.890

Unless otherwise indicated, data presented are the numbers of thoracic MRI scans, with percentages in parentheses. ^a^ Data presented are the medians and interquartile ranges. ^b^ Laboratory data were obtained within 2 weeks of 187 MRI scans in 63 patients (restricted diffusion group: 83 MRI scans in 37 patients; unrestricted diffusion group: 104 MRI scans in 49 patients). ^c^ On-treatment status included cases where the MRI was performed within one week following chemotherapy, radiation therapy, or surgery. Conversely, off-treatment statuses were applied to cases over one week post-treatment, before treatment initiation, or under observation without treatment during the MRI.

## Data Availability

The data presented in this study are available on request from the corresponding author. The data are not publicly available due to ethical restriction.

## Supplementary Materials

The following supporting information can be downloaded at: https://www.mdpi.com/article/10.3390/diagnostics13243654/s1, Figure S1: Illustration of ROI placement for ADC measurement of the thymus.

## Abbreviations

ADC	Apparent diffusion coefficient
DWI	Diffusion-weighted imaging
ROI	Region of interest
