# Supplementary figures and images for: Diffusion-Weighted MR Imaging of the Thymus in Children with Non-Thymic Neoplasms
Source: Diagnostics (Basel). 2023 Dec 13;13(24):3654. doi: 10.3390/diagnostics13243654 (PMC10742587; doi:10.3390/diagnostics13243654)

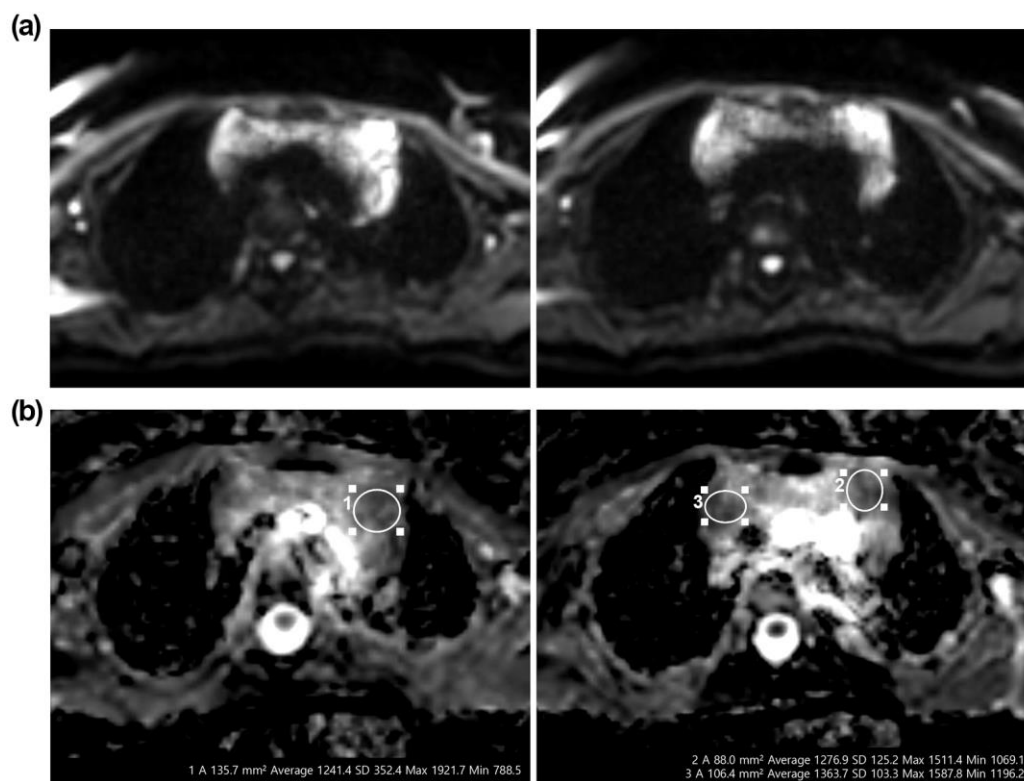

**Figure S1.** Illustration of ROI placement for ADC measurement of the thymus.

Supplement: Supplementary file 1 [file diagnostics-13-03654-s001.zip › diagnostics-2713600-supplementary.pdf]
